# Predicting the naturalistic course of depression from a wide range of clinical, psychological, and biological data: a machine learning approach

**DOI:** 10.1038/s41398-018-0289-1

**Published:** 2018-11-05

**Authors:** Richard Dinga, Andre F. Marquand, Dick J. Veltman, Aartjan T. F. Beekman, Robert A. Schoevers, Albert M. van Hemert, Brenda W. J. H. Penninx, Lianne Schmaal

**Affiliations:** 1 Department of Psychiatry and Amsterdam Neuroscience, Amsterdam UMC, Vrije Universiteit, Amsterdam, The Netherlands; 2 Donders Centre for Cognitive Neuroimaging, Donders Institute for Brain, Cognition and Behaviour, Radboud University, Nijmegen, The Netherlands; 3 Department of Neuroimaging, Institute of Psychiatry, King’s College London, London, United Kingdom; 4 University Medical Center Groningen, Department of Psychiatry, Research School of Behavioural and Cognitive Neurosciences (BCN), University of Groningen, Groningen, The Netherlands; 5 Department of Psychiatry, Leiden University Medical Center, Leiden, The Netherlands; 6 Orygen, The National Centre of Excellence in Youth Mental Health, Melbourne, VIC Australia; 7 Centre for Youth Mental Health, The University of Melbourne, Melbourne, VIC Australia

## Abstract

Many variables have been linked to different course trajectories of depression. These findings, however, are based on group comparisons with unknown translational value. This study evaluated the prognostic value of a wide range of clinical, psychological, and biological characteristics for predicting the course of depression and aimed to identify the best set of predictors. Eight hundred four unipolar depressed patients (major depressive disorder or dysthymia) patients were assessed on a set involving 81 demographic, clinical, psychological, and biological measures and were clinically followed-up for 2 years. Subjects were grouped according to (i) the presence of a depression diagnosis at 2-year follow-up (yes *n* = 397, no *n* = 407), and (ii) three disease course trajectory groups (rapid remission, *n* = 356, gradual improvement *n* = 273, and chronic *n* = 175) identified by a latent class growth analysis. A penalized logistic regression, followed by tight control over type I error, was used to predict depression course and to evaluate the prognostic value of individual variables. Based on the inventory of depressive symptomatology (IDS), we could predict a rapid remission course of depression with an AUROC of 0.69 and 62% accuracy, and the presence of an MDD diagnosis at follow-up with an AUROC of 0.66 and 66% accuracy. Other clinical, psychological, or biological variables did not significantly improve the prediction. Among the large set of variables considered, only the IDS provided predictive value for course prediction on an individual level, although this analysis represents only one possible methodological approach. However, accuracy of course prediction was moderate at best and further improvement is required for these findings to be clinically useful.

## Introduction

Depression is among the leading causes of disability in industrialized countries^1^. Around 20–25% of major depressive disorder (MDD) patients are at risk for chronic depression^2^. To effectively target interventions for patients at risk for a worse long-term clinical outcome, there is a need to identify predictors of chronicity and remission at an early stage. This could allow a quicker escalation of treatment for patients with a low long-term chance of recovery, thus potentially avoiding initial treatment resistance. Chronicity of depression has been linked to various clinical and psychological characteristics, such as the presence of anxiety^2^, longer symptom duration, higher symptom severity, earlier age of onset^3^, and higher neuroticism, lower extraversion and lower conscientiousness^4^. In addition, previous studies have shown that various biological markers including inflammatory markers^5^, lower levels of vitamin D^6^, lower cortisone awakening response^7^, and metabolic syndrome^8^ are associated with a chronicity of depression. The aim of these studies, however, was to find statistically significant group differences, but not to create a predictive model. A statistically significant variable will not necessarily be useful for prediction, due to low effect size or because of its redundancy with respect to other variables. Conversely, even seemingly insignificant variables may become important when combined with other variables. In addition, studies to date have mostly focused on a limited range of potential predictors. It is unknown which (combination) of these many different clinical and biological variables provides the most accurate prediction of naturalistic outcome of depression.

Machine learning (ML)-based predictive models are becoming increasingly more popular for combining large amount of data into one model, and are optimized for evaluating the model’s predictive value for previously unseen individuals (e.g. “new” patients). ML methods have been successfully used to predict MDD persistence, chronicity, and severity^9^, as well as treatment response^10^, suicide attempts of US Army soldiers^11^ and first and new onset of MDD episodes^12,13^. These studies found the most important variables to be severe dysphoria^9^, baseline Quick Inventory of Depressive Symptomatology (QIDS) total severity score^10^, male sex and previous nonviolent weapons offense^11^, lifetime depression screen, and family history^12^. Prediction models in these studies were based on clinical and demographic variables and did not include biological measures.

In the last decades, high hopes have been expressed that the inclusion of biological markers will significantly improve prediction accuracy^9,14^. Biological measures, such as blood and saliva-derived biological measures, may be related to the underlying pathophysiology of depression and therefore may possess prognostic value for disease course^14^. However, currently they are not being routinely used and their efficacy for the prediction is yet to be established.

In the present study, we extended previous studies aimed at identifying predictors of the naturalistic course of depression by including additional psychological and biological predictors and by employing a novel stability selection approach that is designed to select the optimal set of significant predictive variables from a multivariate ML model. We used data from the Netherlands Study of Depression and Anxiety (NESDA), including unipolar depression patients recruited from the community, primary care, and specialized mental health care, thereby capturing a broad range of illness severity^15^. Participants with a depression diagnosis (MDD or dysthymia, *n* = 804) were assessed at baseline and were clinically followed for 2 years. No specific intervention was applied; subjects could have undergone a wide variety of treatments, or no treatment at all. We aim to investigate which variables, among a broad set of clinical, demographic, and psychological variables, as well as biological variables are important and necessary predictors to distinguish depressed patients with a chronic course from patients with more beneficial outcomes over a 2-year course. We focused on the biological variables that have shown to be related to depression or chronicity of depression in the previous cross-sectional studies, including biomarkers of hypothalamic–pituitary–adrenal axis^7^, inflammation^5^, metabolic markers^8^, autonomic nervous system^36^, vitamin D^6^, and neuronal growth factors^32^. We employed ML methods, in combination with a stability selection approach, to identify the optimal set of significant measures that prospectively predict clinical outcome and naturalistic course of depression over 2 years. In addition, we compared the predictive performance of clinical, personality, and biological data modalities. Specifically, we evaluated whether additional data modalities would improve predictive performance of commonly used clinical measures. We employed ML methods, in combination with a stability selection approach, to identify the optimal set of significant measures that prospectively predict clinical outcome and naturalistic course of depression over 2 years.

## Materials and methods

### Participants

Data included in the current study were collected as part of a larger, multi-center study: NESDA. The NESDA aims to study long-term course of depressive and anxiety disorders in a naturalistic cohort study. The sample was recruited from the general population, general practices, and mental health organizations. Subjects were allowed to receive pharmacological or psychotherapeutic treatment or even receiving no treatment at all. The method of recruitment and selection criteria are extensively described elsewhere^15^.

In the present study, we used data from 804 subjects who satisfied additional selection criteria: (i) presence of a DSM-IV MDD or dysthymia diagnosis (or both) in the past 6 months at baseline, established using the structured Composite International Diagnostic Interview (CIDI, version 2.1);^16^ (ii) confirmation of depressive symptoms in the month prior to baseline either by the CIDI or the Life Chart Interview (LCI);^17^ and (iii) availability of 2-year follow-up data on DSM-IV diagnosis and depressive symptoms measured with the LCI. The ethical review boards approved the research protocol and all participants signed written informed consent. Sample characteristics can be found in Table 1.

**Table 1:** Sample characteristics

A: Presence of unipolar depression at follow-up		No	Yes	Statistics	*p*-Value
Sample size *N*		407 (51%)	397 (49%)		
Age		41.07 (12.55)	42.89 (11.83)	*F* = 4.49	0.03*
Male		133 (33%)	145 (37%)	χ2 = 1.15	0.28
Years of education		11.60 (3.17)	11.51 (3.37)	*F* = 0.14	0.71
Antidepressant use baseline		166 (41%)	189 (48%)	χ^2^ = 3.52	0.06
Antidepressant use follow-up		127 (31%)	175 (44%)	χ^2^ = 13.66	0.0002**
Months with antidepressant use between baseline and follow-up		20.58 (25.23)	16.07 (25.67)	χ^2^ = 1.35	0.25
Recruitment type (primary care/specialized care/general population)		162/209/36	143/229/25	χ^2^ = 3.96	0.14
DD/Dysth/MDD diagnosis at baseline		75/16/316	122/18/257	χ^2^ = 17.28	0.0002**
DD/Dysth/MDD diagnosis at follow-up		NA	143/39/215	χ^2^ = 118.33	< 0.0001**
**B: Course trajectory groups**	**Remitted**	**Improved**	**Chronic**	**Statistics**	***p*** **-Value**
Sample size *N*	356 (44%)	273 (34%)	175 (22%)		
Age	40.60 (12.57)	42.36 (12.29)	44.13 (11.07)	*F* = 5.16	0.01**
Males	109 (31%)	97 (36%)	72 (41%)	χ^2^ = 5.91	0.05*
Years of education	11.70 (3.15)	11.40 (3.2)	11.51 (3.59)	*F* = 0.66	0.52
Antidepressant use baseline	139 (39%)	120 (44%)	96 (55%)	χ^2^ = 11.90	0.0026**
Antidepressant use follow-up	112 (31%)	106 (39%)	84(48%)	χ^2^ = 13.97	0.0009**
Months with antidepressant use between baseline and follow-up	21.9 (29.37)	13.99 (12.35)	20.02 (33.37)	χ^2^ = 1.66	0.19
Recruitment type (primary care/specialized care/general population)	147/178/31	101/155/17	57/105/13	χ^2^ = 6.26	0.18
DD/Dysth/MDD diagnosis at baseline	56/13/287	78/8/187	63/13/99	χ^2^ = 38	< 0.0001**
DD/Dysth/MDD/No diagnosis at follow-up	2/1/85/268	73/22/71/107	68/16/59/32	χ^2^ = 223.42	< 0.0001**
**C: Correspondence of the outcome definitions**	Course trajectory groups				
Presence of unipolar depression at follow-up		**Remitted**	**Improved**	**Chronic**	
	No	268 (75%)	107 (39%)	32 (18%)	
	Yes	88 (25%)	166 (61%)	143 (82%)	

Data are given as mean (SD) or *N* (%)

The table shows characteristics of the sample divided by two outcome definitions: (A) Presence or absence of a unipolar depression diagnosis (major depressive disorder or dysthymia) 2 years after baseline measurement. (B) Three course trajectories derived from a latent class growth analysis on burden of depressive symptoms indicated for each of the 24 months between baseline and follow-up: a rapid remission, gradual improvement, and a chronic course. Duration of antidepressant use is measured in months between baseline and 2-year follow-up. SD; standard deviation. (C) Overlap of outcome groups

*MDD* major depressive disorder, *Dysth* dysthymia, *DD* double depression (MDD + dysthymia), *p ≤0.05, **p ≤0.01 two-tailed

### Definition of outcome groups

We defined outcome groups in two ways: (i) based on the presence or absence of a current unipolar depression diagnosis (6-month recency MDD diagnosis or dysthymic disorder) at 2-year follow-up, according to DSM-IV MDD criteria and (ii) groups based with different trajectories of burden of their depressive symptoms over a 2-year period following baseline derived from a latent class growth analysis (LCGA) conducted previously in the same sample^18^. The LCGA identified five different course trajectory groups: a rapid remission trajectory, two groups with a trajectory showing a gradual improvement of symptoms that differ in initial severity of depressive symptoms, two chronic trajectories (one with moderate initial severity and the other with severe initial severity). Because the two improving trajectories, as well as the two chronic trajectories were similar in terms of trajectory of symptoms (they differed only in initial symptom severity at baseline) and for the purpose of increasing statistical power, we combined these pairs, yielding three course trajectories: (1) remission (REM), showing a rapid remission of symptoms (*n* = 356); (2) improving (IMP), showing a gradual improvement in symptoms from baseline to follow-up (*n* = 273); and (3) chronic (CHR), showing no relief from symptoms from baseline to follow-up (*n* = 175). See Rhebergen et al.^18^ and supplemental material for detailed information about the LCGA procedure.

### Baseline predictor variables

#### Clinical variables

We included 55 clinical variables as predictor variables, including measures of depressive symptoms, as indicated by the summary score of the inventory of depressive symptomatology (IDS) questionnaire^19^. Diagnostic information on MDD, dysthymia, and anxiety-related measures were derived from the CIDI^16^. The summary score of anxiety severity was measured using the Beck Anxiety Inventory (BAI)^20^. Childhood trauma (before the age of 16) was measured with a childhood trauma interview as used in de Graaf et al.^21^ and family history (presence of a first-degree family member with MDD or anxiety) was measured using the family tree method^22^. Additional information about variable scoring and collection can be found in supplemental materials.

#### Psychological traits

We included five personality dimensions as predictor variables, including neuroticism, extraversion, openness to experience, agreeableness, and conscientiousness, measured with the NEO five-factor inventory^23^. Each dimension was measured by 12 items scored on a five-point Likert scale.

#### Demographic variables

Age, gender, and number of years of education were included as predictor variables.

#### Biological variables

We included general measures of somatic health including body mass index, waist circumference, lung-capacity, hand-grip strength, and number of chronic somatic diseases under treatment. Inflammatory markers included C-reactive protein (CRP), interleukin-6 (IL6), and tumor necrosis factor-alpha. Metabolic syndrome variables included triglyceride level, high-density lipoprotein cholesterol level, systolic and diastolic blood pressure, and fasting glucose level. Metabolic syndrome variables were adjusted for medication use. Mean heart rate and heart rate variability during interview were used as measures of autonomic nervous system. We also included measures of vitamin D, brain-derived neurotrophic factor (BDNF), and cortisol. The details of data collection procedures can be found in supplemental materials.

### Statistical analysis

#### Prediction of MDD diagnosis at follow-up and trajectory course groups

We used penalized (elastic-net) logistic regression from the R package glmnet^24^ to predict the presence or absence of a unipolar depression diagnosis at 2-year follow-up and its multinomial generalization to predict the three LCGA course trajectory groups. The elastic-net penalty allows building a sparse model, thereby performing feature selection (for details, see supplemental materials). To assess generalizability, we performed 10-fold cross-validation, repeated 10 times. For each of 10 repetitions, the complete dataset was divided into 10 equally sized subsamples, of which 9 were used as a training set to create a model and the 10th was used as a test set. To quantify generalization error, we measured the area under the receiver operating curve (AUROC, the proportion of times a randomly selected subject from a positive class is ranked before a randomly selected subject from a negative class), sensitivity, specificity, balanced accuracy (mean of sensitivity and specificity), and positive and negative predictive value. For multinomial predictions, we assessed the same performance measures for predicting each group separately from the other two (referred to as a “one vs. all” configuration in the ML literature). We also assessed mean sensitivity (mean of proportion of correctly classified subjects in each group) as a multi-class version of balanced accuracy. We used balanced accuracy and mean sensitivity instead of accuracy to accommodate unequal group sizes. Permutation testing was used to determine statistical significance (see supplementary materials for more details). We conducted additional exploratory analyses to detect potential interaction or nonlinear effects by testing additional models that include all two-way interaction terms and a polynomial expansion of age. A description of the statistical procedure and the results of these exploratory models can be found in supplementary materials.

#### Identification of discriminating variables

Variable selection is well known to be a difficult problem in settings where the predictor variables are highly collinear (as they are here). Specifically, the variables detected can be highly sensitive to slight variations in the data and it can be difficult to determine whether variables are selected because they are directly useful in predicting the outcome or because they help canceling out noise or mismatch in other covariates^25^. To address this issue, we used a stability selection approach^26^ that finds a stable set of features that predicts the outcome and provides tight family-wise error control over the number of falsely selected variables (type I error rate). Specifically, the model is fitted many times on different subsamples of the data, to estimate the chance of each variable to be selected. Given a specified selection threshold (e.g., selection threshold of 0.75 means that a variable has a 75% chance of being selected, or in other words, the variable is selected in 75% of the subsamples of the data, see supplementary materials), stability selection theory, derived from Meinshausen et al.^26^, provides a particular family-wise error bound on the expected number of falsely selected features at each point along a “stability path” that tracks the variables included in the model as a function of regularization strength. These stability paths are also a useful tool for visualization and show the region on the stability path where the probability of a false selection is sufficiently low. To perform stability selection, we used the R package C060^27^.

## Results

Demographic and clinical characteristics of the two follow-up diagnosis groups and three LCGA course trajectory groups can be found in Table 1.

### Prediction of the presence of an MDD diagnosis at 2-year follow-up

**Fig. 1:**
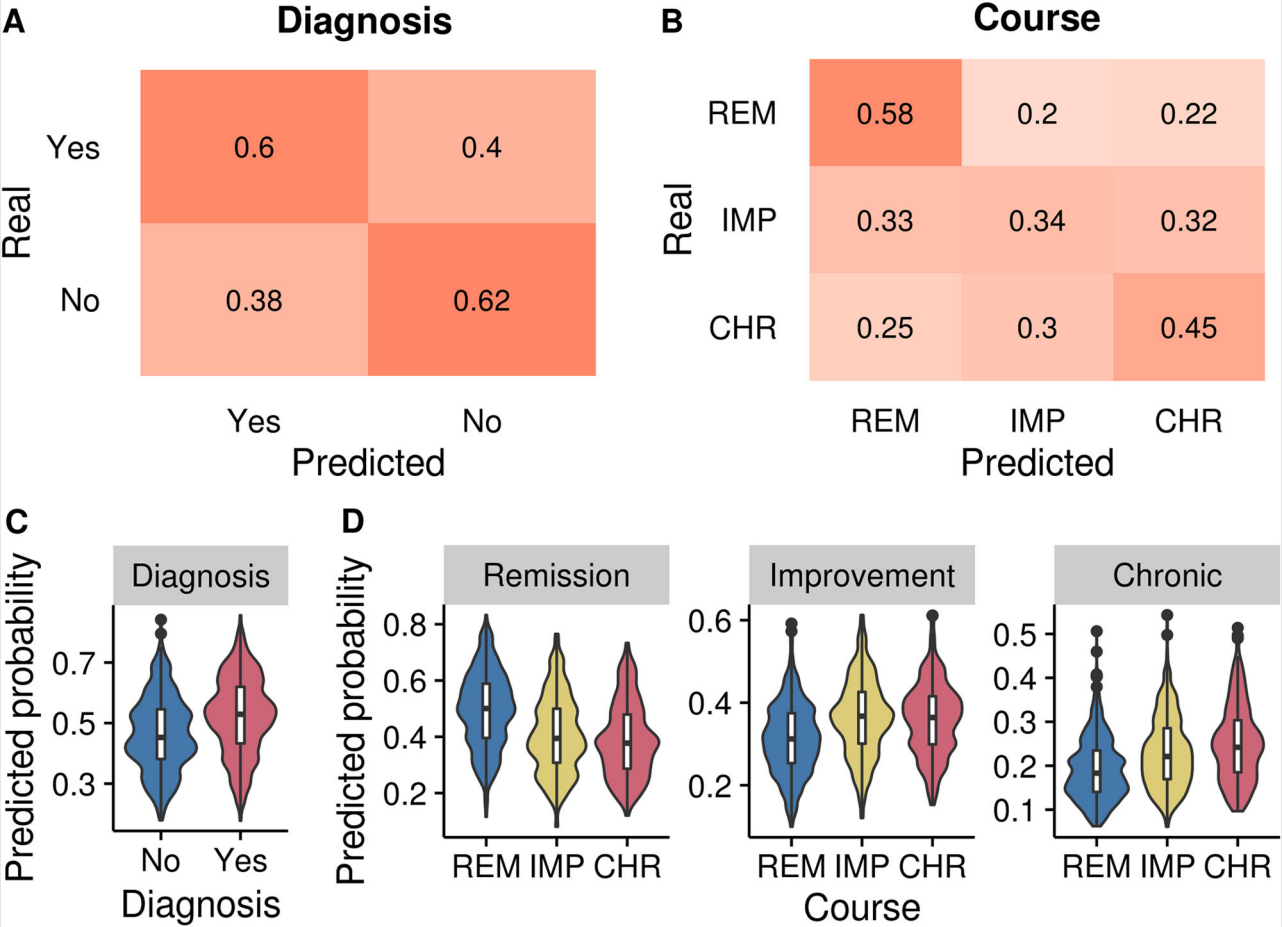
Model predictions. Confusion matrices for classifiers are depicted in panel **a** for binary prediction, i.e., presence or absence of a unipolar depression diagnosis at follow-up (major depressive disorder or dysthymia), and **b** for prediction of the three LCGA course trajectory groups. Number and color in each cell describe the proportion of predictions. For example, chance level would be 0.5 in each cell in the confusion matrix in **a**, and 0.333 in the confusion matrix in **b**. Violin plots of the spread of predicted values are depicted in panel **c** for binary prediction, i.e., presence or absence of a unipolar depression diagnosis at follow-up, and **d** for predicting the three course trajectory groups

The penalized logistic regression trained on all demographic, clinical, psychological, and biological predictors discriminated between patients with and without a unipolar depression diagnosis at 2-year follow-up with 0.66 AUROC and 62% balanced accuracy. The confusion matrix is shown in Fig. 1a and the spread of predicted outcomes in Fig. 1c. Graphs depicting positive and negative predictive values can be found in supplementary materials (Figures S2, S3).

### Prediction of LCGA course trajectory groups

Using all clinical, psychological, and biological predictors, we could discriminate between the three course trajectory groups; rapid REM with 0.69 AUROC and 66% balanced accuracy, the gradual IMP group with 0.62 AUROC and 60% balanced accuracy, and the CHR group with 0.66 AUROC and 61% balanced accuracy. In the case of multinomial prediction, sensitivity for each group was 59% for REM, 37% for IMP, and 47% for CHR (chance level with three groups is 33%). The confusion matrix for the multinomial prediction is shown in Fig. 1b and the spread of predicted outcomes in Fig. 1d. The average sensitivity of all three groups was 0.47, which was significantly higher than a chance level of 0.33 (*p* < 0.05). Graphs depicting positive and negative predictive values can be found in supplementary materials (Figures S2, S3).

### Identification of discriminating variables

**Fig. 2:**
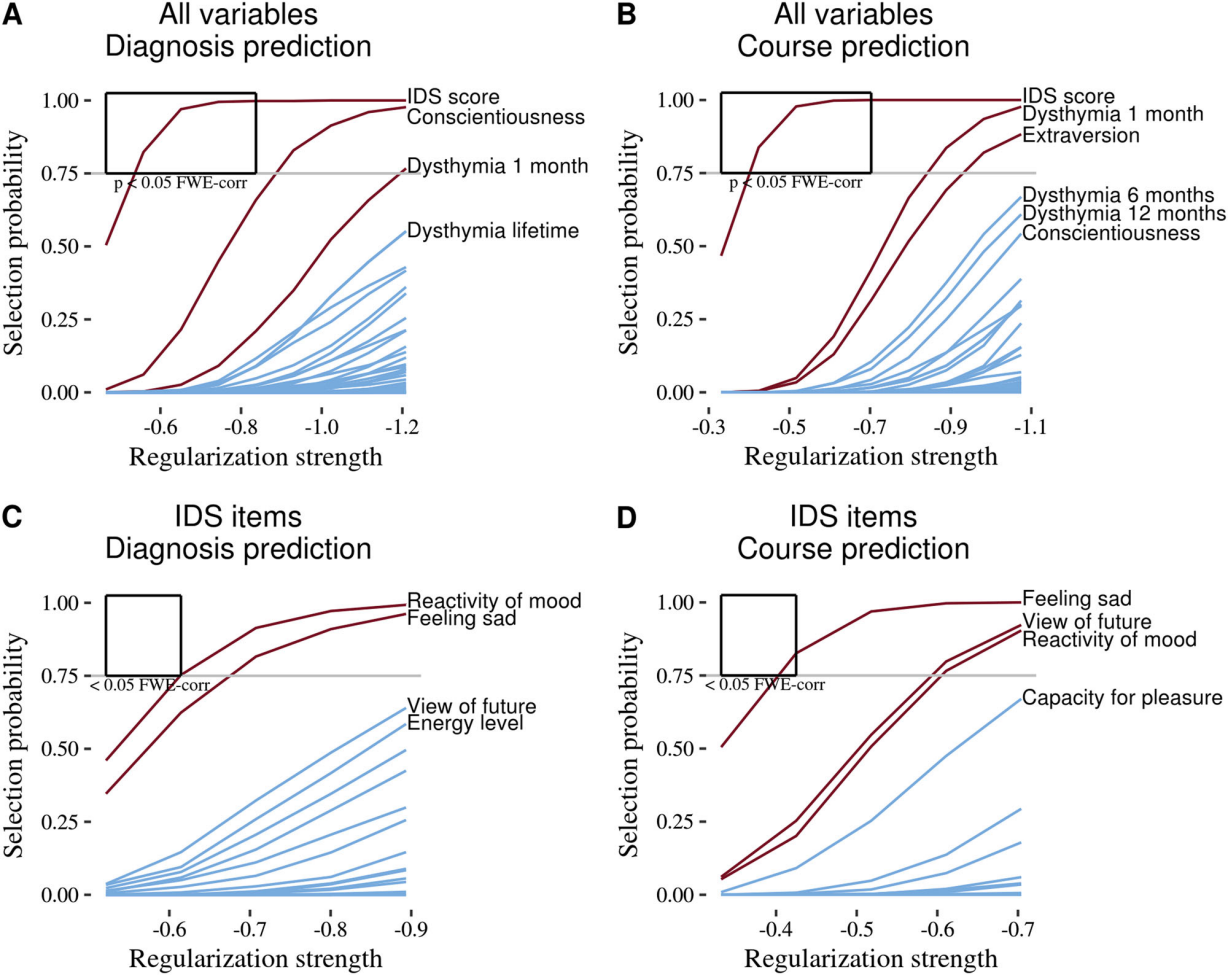
Stability paths. Stability paths of elastic-net logistic regression showing selection probabilities of each variable with respect to amount of applied regularization. The less regularization is applied, the more variables will be included in the model and the higher the chance for a false-positive selection. The stability selection approach allows us to statistically control for false-positive discovery. Variables crossing the marked regions are statistically significantly related to the outcome variable with the error correction *p*_fwer_ < 0.05 according to the stability selection theory. Other variables that crossed the probability threshold (they have been selected at least 75% of times under resampling) might also be important, but they did not survive the multiple comparison correction. **a**, **b** Logistic regression trained on all variables. **c**, **d** Logistic regression trained only on the individual items from the inventory of depressive symptomatology (IDS) questionnaire

Figure 2a, b show stability paths indicating how often each variable in the model is selected as a function of the regularization applied. The IDS total score is the only variable that survived family-wise error correction (with *p*_fwer_ < 0.05), both for predicting outcomes defined as the three LCGA groups and as the presence of a unipolar depression diagnosis at follow-up. Also, IDS score was selected much sooner in the stability path than other variables, indicating a high probability of the IDS score being included in the model, even if that model would contain a minimal number of variables. To examine the direction of effect of stable predictors, we fitted a model including only the first nine variables that cross the selection threshold. The coefficients and univariate correlations of these variables are in Table 2. The direction of the effects of clinical variables is as expected, the presence of dysthymia or suicidality decrease the chance of a better outcome.

**Table 2:** Coefficients of selected variables

A:	Presence of a unipolar depression diagnosis at follow-up						
**Rank** ^**a**^		**β** ^**b**^	**r**_**pb**_^**c**^				
	(Intercept)	−0.03	—				
1	IDS score^d^	0.39	0.25				
2	Conscientiousness	−0.33	−0.19				
3	Extraversion	−0.04	−0.16				
4	Neuroticism	−0.06	0.16				
5	MDD criteria^e^	0.1	0.14				
6	Dysthymia lifetime	−0.13	0.15				
7	Dysthymia 1m^f^	0.19	0.16				
8	Dysthymia	0.2	0.15				
9	Mild recurrent MDD	−0.11	−0.13				

**B:**	**Course trajectories**	**Remitted**		**Improved**		**Chronic**	
**Rank** ^**a**^		**β** ^**b**^	**r**_**pb**_^**c**^	**β** ^**b**^	**r**_**pb**_^**c**^	**β** ^**b**^	**r**_**pb**_^**c**^
	(Intercept)	0.31	—	0.09	—	-0.4	—
1	IDS score^d^	−0.31	−0.29	0.12	0.16	0.19	0.16
2	Conscientiousness	0.13	0.16	−0.08	−0.11	−0.04	−0.07
3	Extraversion	0.09	0.2	−0.05	−0.12	−0.04	−0.11
4	Suicidality	−0.1	−0.15	0.1	0.11	0	0.05
5	Dysthymia lifetime^f^	0.14	−0.16	−0.04	0.02	−0.1	0.16
6	Dysthymia 12m^f^	−0.04	−0.18	−0.04	0.04	0.09	0.17
7	Dysthymia 6m^f^	0.24	−0.18	−0.04	0.04	−0.2	0.17
8	Dysthymia 1m^f^	−0.41	−0.2	0.15	0.06	0.26	0.18
9	Dysthymia	−0.16	−0.16	−0.05	0.02	0.22	0.16

^a^Features are ranked based on order of selection by the stability selection approach

^b^Coefficients of the logistic regression models. In the case of a multi-class problem (table B), coefficients of each of the binary regressions are shown. However, the direction and a magnitude of coefficients are hard to interpret due to a collinearity problem

^c^Univariate (point biserial) correlation coefficients showing the relationship of individual variable with different course groups

^d^IDS, inventory of depressive symptomatology

^e^Number of DSM-IV diagnostic criteria met for a diagnosis of major depressive disorder (MDD)

^f^Recency of dysthymia in months

Other variables that were selected but did not survive FWE (family-wise error rate) correction included: dysthymia diagnosis (1-month recency) and conscientiousness for the prediction of the presence of a unipolar depression diagnosis at follow-up, and a dysthymia diagnosis in the past 1 and 6 months, as well as extraversion for discriminating between the three LCGA course trajectory groups.

### Predictive performance of individual predictor domains

**Fig. 3:**
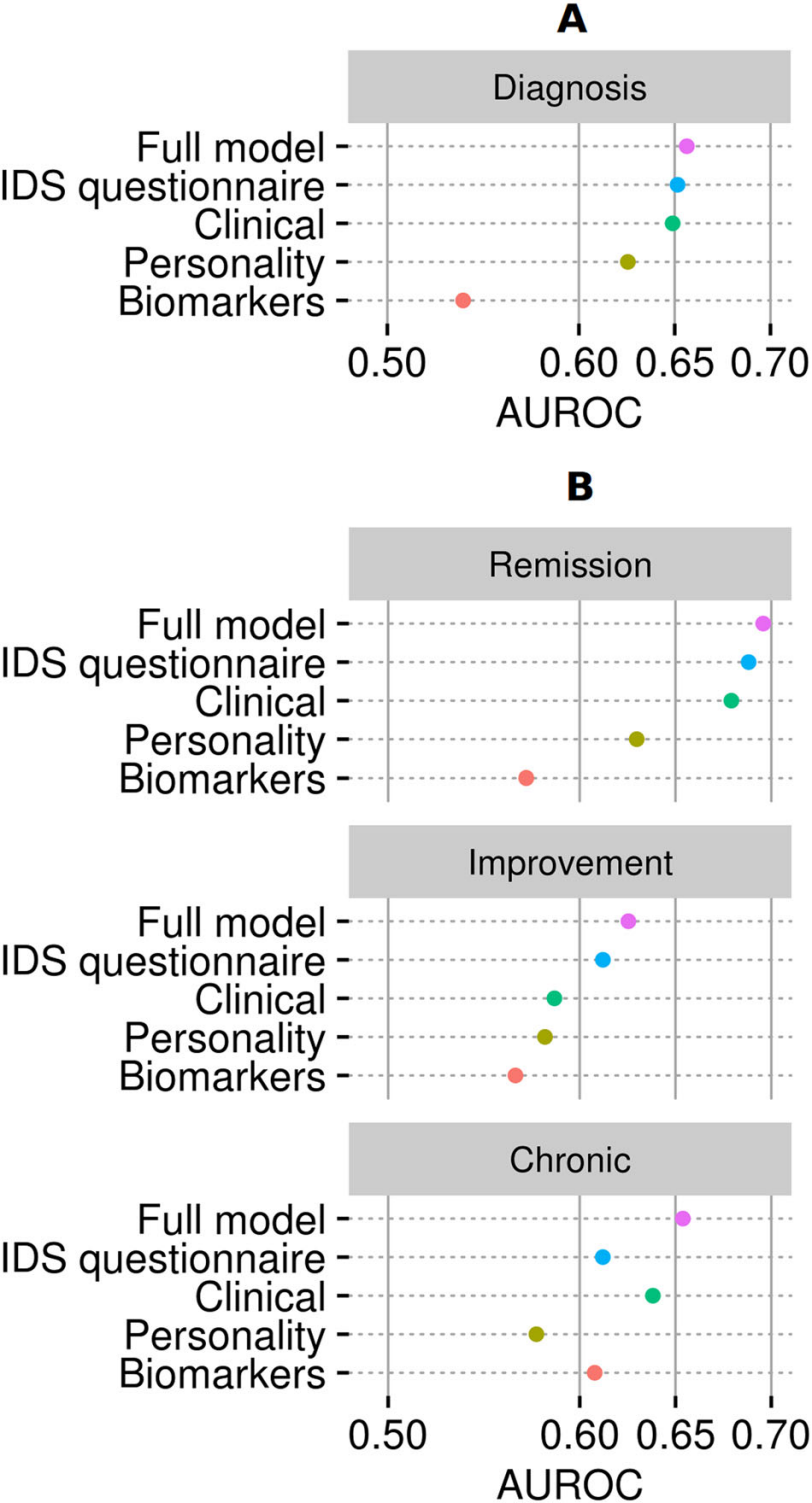
Performance of different data modalities. Mean area under the curve for predictive models of naturalistic course of depression. **a** Predicting the presence or absence of a unipolar depression diagnosis 2 years after the baseline measurement. **b** Predicting the three depression course trajectory groups

We compared performance of individual predictors domains, including (i) IDS items, (ii) 55 clinical measures, (iii) 5 psychological measures, and (iv) 18 biological measures. Across all outcomes, the model using all variables performed better than predictors within individual domains. Best performance was observed for prediction of the REM group. With regard to individual predictor domains: prediction based on IDS item scores showed the best prediction. The prediction using only biological variables showed the lowest performance for three out of four outcomes, and they could only significantly discriminate the CHR group. The performance of the IDS item model was within 0.01 AUROC of the performance of the full model (including all predictor variables) for REM and IMP outcomes and the presence versus absence of an unipolar depression diagnosis after 2 years (Fig. 3). The only exception was a decrease of model performance using only the IDS items for discriminating the CHR group from the other two LCGA groups; performance dropped from 0.66 (full model) to 0.61 (IDS items only) AUROC. The models trained on all clinical, psychological, and biological variables separately, showed lower AUROC values compared with the IDS item model and the full model for discriminating REM and IMP groups. In case of CHR group, clinical variables were more predictive than IDS items alone (Fig. 3b). Psychological measures discriminated significantly better than chance the REM group and presence of a unipolar depression diagnosis at follow-up. Clinical measures discriminated significantly between all groups except the IMP group.

### Predictive performance of individual IDS items

As only the IDS total score was statistically significant, we examined which items of the IDS contributed most to this prediction. We performed post-hoc stability selection analyses including only individual IDS item scores. From 30 items, only the item “Feeling sad” was selected as a statistically significant predictor (with *p*_fwer_ < 0.05) for discriminating between the three LCGA groups (Fig. 2c). For predicting the presence of a unipolar depression diagnosis at follow-up, the item “Feeling sad” was also selected, but did not survive the FWER correction. Instead, mood reactivity was statistically significant (Fig. 2d).

## Discussion

Our findings indicate that from a wide range of clinical, biological, and psychological predictors, only severity of baseline depressive symptoms (measured by the IDS self-report questionnaire) was a significant predictor of different course trajectories of depression. We were able to predict the presence or absence of a unipolar depression diagnosis after 2 years with an AUROC of 0.66, and to discriminate between three course trajectory groups with an AUROC of 0.69 for rapid REM, 0.63 for gradual IMP, and 0.66 for a CHR course of depression.

Prediction of outcome in psychiatry is notoriously hard, due to heterogeneity of disorders, broad comorbidities across disorders, and due to clinical categories defined without a priori biological validity^28^. The performance of our models will need to improve in order to be translatable to clinical practice, but is comparable to previous ML studies predicting the *naturalistic course* of depression. For example, a study by Kessler and colleagues^9^ observed an AUROC of 0.63 for predicting high chronicity, defined as an episode lasting most days throughout the year, and AUROC’s between 0.71 and 0.76 for predicting other measures indicative of a 10- to 12-year illness course of depression, such as high persistence of MDD, hospitalization, and disability caused by MDD, and suicide attempts. Kessler et al.^9^ based their prediction models on baseline clinical measures alone, including symptoms of MDD and parental history of MDD, mania–hypomania, anxiety disorders, and externalizing disorders. The contribution of each individual clinical measure to the overall prediction was not assessed, so we cannot infer whether the prediction of their outcomes was also driven by severity of symptoms as observed in the present study.

Our most accurate models achieved a slightly lower AUROC of 0.69 for predicting an illness course characterized by rapid remission compared with the AUROCs found in Kessler et al.^9^, which is arguably less extreme (and therefore likely harder to predict) and a more prevalent outcome than outcomes considered by Kessler and colleagues^9^. Highest AUROC’s were found by Kessler et al.^9^ for models predicting hospitalization, disability, and attempted suicide, which was reported in only 3.2–5.8% of the total sample. However in our study, the prevalence of a remitted course of depression was 44%. Therefore, despite the smaller AUROCs, the positive predictive value (PPV) of our models is higher (between 33 and 68% PPV for a given outcome definition in the present study (Supplemental Figure S2), compared with PPV between 12.5% and 18.3% in the Kessler et al. study^9^ for 20% of subjects with highest predicted probability of a given clinical outcome), which means that our models have a smaller probability of false-positive classifications. Previous studies aimed at predicting *first onset* and *new onset* of an MDD episode during follow-up in people with no current MDD diagnosis using prediction algorithms based on demographic and clinical characteristics have found slightly higher AUROC of 0.75–0.79 in the training sample, which dropped to 0.70–0.73 AUC in independent replication samples^12,13,29^.

In addition to evaluating the predictive value of a model including all clinical, biological, and psychological variables, we aimed to identify (a minimum set of) individual predictors that reliably predict the naturalistic course of depression. For this purpose, we used a novel approach by combining penalized logistic regression and a stability selection method that selects predictive variables from a multivariate model while controlling for family-wise error. Of all included measures, we only identified the IDS as a statistically significant predictor. The total IDS score was positively associated with IMP and CHR course group membership, and with the presence of a depression diagnosis after 2 years, and negatively associated with a REM course and the absence of a depression diagnosis at follow-up. Although our method provides excellent control over type I errors, it is conservative and can miss predictive variables^26^. However, other variables only improved prediction of CHR course by 0.05 AUROC and all other outcome groups (presence of an MDD diagnosis, REM, IMP) by 0.01 AUROC. This is roughly equivalent to a difference of 0.04 Cohen’s *d*^30^, indicating a low added value of additional variables over and above the IDS. Interestingly, a subsequent exploratory analysis that we performed to identify which individual items of the IDS contributed most to the prediction showed that only the items “Feeling sad” (for predicting the presence of an MDD diagnosis at follow-up) and “Response of your mood to good or desired events” (for predicting the three different course trajectories) were identified as significant predictors. Performance of models using only these two items was similar to a model using all IDS items.

Similar results were found by Chekroud and colleagues^10^ in a recent study examining the predictive value of clinical measures for remission of MDD symptoms following a randomized 12-week citalopram treatment. Their model selected 25 best predictors from 164 sociodemographic and clinical features, and was able to predict remission with AUC of 0.70. Total severity of depressive symptoms, measured with the QIDS (shortened version of the IDS) was the most important predictor of treatment response. In line with the current study, treatment response could also be predicted with models using fewer variables, e.g., with only 15 and 10 variables with AUC of 0.69 and 0.68, respectively.

These findings suggest that other clinical measures possess very little or no prognostic value for course of depression—or remission following treatment in the Chekroud et al. study^10^—above and beyond severity of depressive symptoms. Biological variables, including inflammatory markers, cortisone, metabolic measures, BDNF, and vitamin D were able to predict only a chronic course of depression, although performance was worse than for clinical variables. This finding is in contrast to our previous studies within the same sample that showed group-level associations between lower cortisol awakening response^7^ and vitamin D deficiency^6^ and chronicity of unipolar depression. These findings show clearly that a group-level association does not imply the ability to make predictions for new cases at the level of individual subjects. This implies that although these baseline biological parameters can be associated with outcome based on group-level approaches, the effect sizes are probably too small to possess sufficient prognostic ability for long-term outcome in individual patients. In line with the current findings, in previous studies we found no group-level associations between a chronic course of depression and BDNF^31^, CRP, IL6 and metabolic syndrome^32^, despite clear group differences between healthy controls and unipolar depression patients. This may suggest that biological markers implicated in the etiology of unipolar depression are not necessarily good prognostic markers. Nonetheless, although we found no evidence for biological variables being informative for predicting *naturalistic course* of depression at the level of individual patients, they may still be useful for discriminating unipolar depression patients from other patient groups, e.g., bipolar disorder^33^, or for predicting response to, e.g., antidepressant treatment. Moreover, our course outcome definitions were based on DSM diagnosis and severity of symptoms. Symptom-based classifications are agnostic about underlying biological mechanisms and patients whose trajectory of symptoms is caused by different biological processes may be subsumed under the same category. As a consequence, our different course trajectory groups may have consisted of a heterogeneous set of patients with a similar course in terms of symptoms but distinct underlying pathophysiological mechanisms, and, hence, the full predictive power of biological variables may become only visible when patients are first stratified according to clinically relevant biological characteristics.

We previously showed promising results using task-based functional brain imaging^34^. This study was conducted in a smaller subsample (*n* = 118) of the dataset used here, with identical LCGA course trajectory definitions. In this study, models based on neural patterns of activation in response to emotional facial expressions could discriminate chronic patients from patients with more favorable trajectories with up to 73% accuracy and outperformed models based on other neuroimaging modalities (structural magnetic resonance imaging, task-based functional magnetic resonance imaging related to executive functioning with a chance level accuracy) or clinical data (accuracy of 69%). However, since the sample in our previous study was smaller, resulting in less stable results, and more homogeneous due to additional selection criteria, no strong conclusions can be drawn regarding the added value of neuroimaging data.

### **Limitations**

The main limitation of the current study is a lack of replication of our findings in an independent dataset. Although within-sample cross-validation is known to be an approximately unbiased estimator of population generalizability^35^, it may not completely account for the different characteristics of data from different samples. An important next step is to validate our findings in independent data. An additional limitation is that due to the naturalistic setting of our study treatment was not controlled and limited information was available on treatment received during the follow-up period. The advantage of our naturalistic design is that the sample is more representative of depression in the general population. However, the prediction accuracy may have been higher in a more homogeneous and controlled sample. A final limitation of the study is that we tested only a one ML algorithm without the extensive tuning of all hyper-parameters. It is possible that a different analytic pipeline or an algorithm would yield slightly different predictions. We have done this mainly for the sake of simplicity so that stability selection is performed on the same algorithm that was also used to make predictions, and to avoid overly optimistic results due to model selection bias and overfitting^36^. Our results can, therefore, be considered as a conservative estimate of out of sample predictive accuracy.

## Conclusion

The current study showed that for prediction of the naturalistic course of depression on the level of individual patients, only severity of depressive symptoms was identified as a stable and significant predictor with low to moderate prediction accuracy. Among a wide set of psychological, biological, and clinical variables no other measure improved the prediction accuracy that was obtained based on self-reported depressive symptoms (IDS scores) alone. However, our best model only showed moderate predictive performance at best, hence, the prediction model requires further improvements to be clinically useful.

## Electronic supplementary material

Supplemental material
