# Supplementary material for: Predicting the naturalistic course of depression from a wide range of clinical, psychological, and biological data: a machine learning approach
Source: Transl Psychiatry. 2018 Nov 5;8:241. doi: 10.1038/s41398-018-0289-1 (PMC6218451; doi:10.1038/s41398-018-0289-1)
Supplement: Supplementary file 1 — Supplemental material [file 41398_2018_289_MOESM1_ESM.pdf]

Predicting the Naturalistic Course of Depression From a Wide Range of Clinical, Psychological and Biological Data: A Machine Learning Approach

Supplemental Information

Supplemental methods

Baseline predictor variables

Clinical variables

Clinical predictors included measures of depressive symptoms, as indicated by the summary score of the Inventory of Depressive Symptomatology questionnaire (IDS).[1] MDD and dysthymia related measures were derived from the CIDI: presence of each of the 9 individual MDD DSM-IV criteria (yes/no score for each of the 9 MDD criteria), severity of MDD (yes/no mild, moderate, severe), recency of MDD (4 yes/no indicators for presence of MDD in past 1 month, 6 months, 12 months and life-time), age of onset of MDD, presence and recency of dysthymia (4 yes/no indicators for presence of dysthymia in past 1 month, 6 months, 12 months and life-time), recurrence of MDD (yes/no first episode), and number of prior MDD episodes. In addition, measures of anxiety included the summary score of anxiety severity measured by the Beck Anxiety Inventory (BAI);[2] the presence or absence of each of the following DSM-IV anxiety disorders assessed by the CIDI: social phobia, agoraphobia, panic disorder, panic agoraphobia and general anxiety disorder, recency of each of the anxiety disorders (yes/no presence of each disorder in 1, 6 and 12 months and lifetime), as well as the total number of current anxiety diagnoses.

Childhood trauma (before the age of 16) was assessed using a childhood trauma interview as used in De Graaf et al.[3] The total childhood trauma score was calculated as the sum (0-8) of number and frequency of traumatic events scored 0-2 for 4 domains: emotional neglect, psychological abuse, physical abuse and sexual abuse (for more details see [4]).

Family history was evaluated using the family tree method.[5] Persons with a first-degree family member with depression or anxiety were considered to have a positive family history.

Biological variables

Waist circumference was measured using a measuring tape at the central point between lowest front rib and highest point of pelvis. Pregnant women were not measured. Vitamin D and inflammatory markers CRP, IL6 and TNF-alpha were measured from fasting blood samples, collected in the morning of baseline interview and kept frozen at -80c. Detailed information about assessment of vitamin D and inflammatory markers are provided elsewhere.[6] Cortisol levels were

assessed from saliva samples using Salivettes (Starstedt AG, Numbrecht, Germany). Participants were instructed to collect saliva samples during a regular (working) day. We used two first-hour morning awakening curve cortisol measures integrated in an area under the curve with respect to the ground (ROCg) and with respect to the increase (ROCi), as these best reflect the HPA-axis hyperactivity and have shown to be strongest associated with depression in our study (for more details, see [7]). BMI was calculated as measured weight in kilograms divided by squared height in meters.

We also included indicators of physical fitness, namely hand-grip strength and lung function. Hand-grip strength was measured by a Jamar hand-held dynamo-meter, during a sitting, straight backed position. Lung function was measured as a peak expiatory flow using a mini Wright peak flow meter. Details of those procedures are described in Van Milligen et al.[8]

The total number of chronic diseases was established based on a self-report questionnaire, and included lung disease, diabetes, cardiovascular disease, cancer, osteoarthritis, intestinal disorder, liver disease, epilepsy and thyroid gland diseases for which a respondent received treatment and/or medical attention.

Heart rate and heart rate variability were assessed using the ‘Vrije Universiteit Ambulatory Monitoring System” (VU-AMS) during the large time of the baseline assessment; medical examination, interview, and a computer task (approximately 100 minutes). ECG was assessed using a six-electrode configuration. Non-stationary periods measured by an accelerometer were discarded. Additional parts of the recording were discarded upon visual inspection.

Definition of outcome groups using Latent Class Growth Analysis

We used a 3 class outcome definition based on course trajectories of burden of depressive symptoms identified previously in the same sample[9]. The burden of depressive symptoms was assessed using the Life Chart Inventory[10] assessed at 2-year follow-up. The Life Chart Inventory retrospectively assessed the burden of depressive symptoms for each month during the previous 2 years (between baseline and follow-up). The occurrence of life events was explored first, to refresh the memory, and then the presence of depressive symptoms and their self-reported burden was assessed using a 5-point likert scale. This resulted in 24 scores on which the Latent Class Growth Analysis (LCGA) was applied. The LCGA identified 5 distinct course trajectories that differed both in initial severity and change of symptom burden over time (Figure S1). Because LCGA is known to oversplit the data (find more categories than there really are)[11] and to have a sufficient number of subjects in each course trajectory group, we decided to combine the categories with similar course (slope) but a different initial severity into 3 final outcome groups: rapid remission, gradual improvement and chronic.

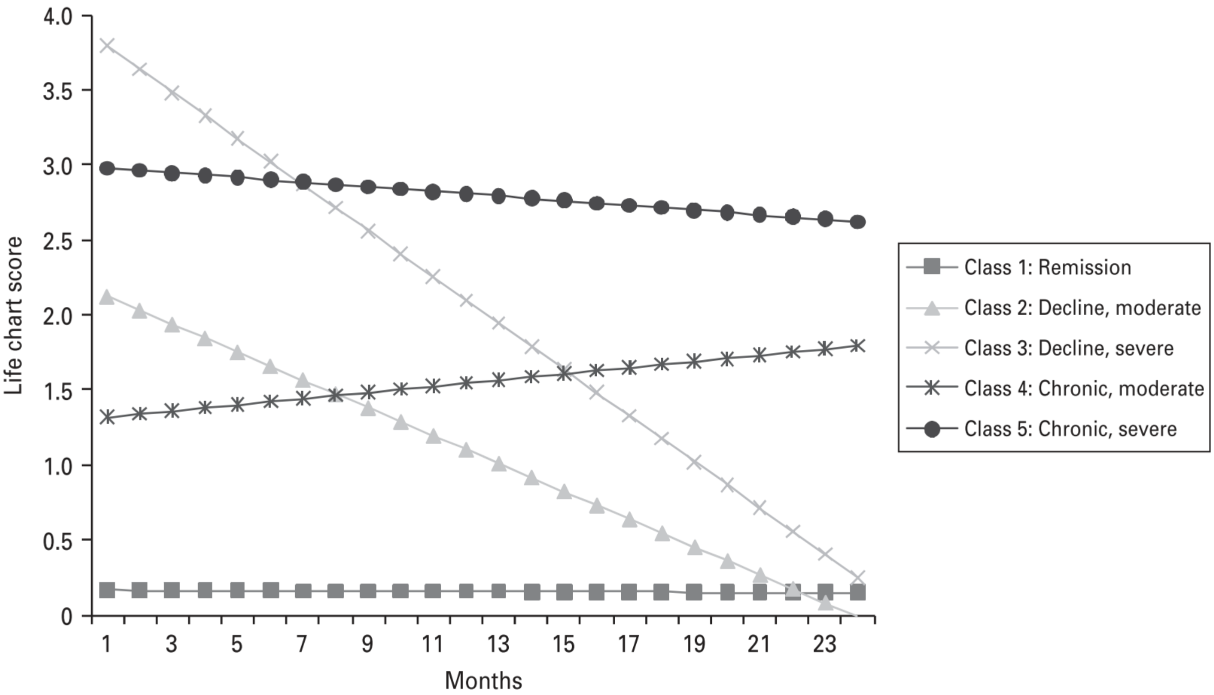

Figure S1: Course trajectories identified in Rhebergen et. al.. For the current study, we combined the 5 course trajectories into 3 by merging both decline categories together and both chronic categories together.

Statistical analysis

In order to combat high correlations between our variables and to prevent over-fitting and thus low out of sample prediction, we used logistic regression with an elastic net penalty. Penalization is achieved by adding additional penalization term into a loss function measuring data fit. The addition of the penalization term aims to create simpler - and therefore potentially less overfit - models. We used an elastic net penalty[12] that linearly combines two other commonly used methods for penalization: lasso and ridge penalization.

More specifically, we used a penalized binomial and multinomial logistic regression. The method is explained in detail elsewhere.[13] In brief, multinomial logistic regression is an extension of binomial logistic regression to K>2 classes. Since the binomial logistic regression can be seen as a special case of multinomial logistic regression with K=2 classes, here we will describe only the multinomial version. Multinomial logistic regression models the probability of subject *i* belonging to class (i.e. course trajectory group) ℓ with a softmax function:

$$Pr\left(G=l \vee x\right)=\frac{e^{\beta_{0l}+x_i^T \beta_l}}{\sum_{k=1}^{K} e^{\beta_{0k}+x_i^T \beta_k}}$$

where $\beta_{0\ell}$ and β_ℓ are respectively the intercept and regression coefficients for the logit function modeling the ℓ-th class, K is the number of classes and x_i^T is a vector of predictor variables for the i-th sample.

This model is fit by maximizing the penalized multinomial log-likelihood function:

$$f\left(\{\beta_{0l},\beta_l\}_1^K\right)=\frac{1}{N}\sum_{i=1}^{N}\left[\sum_{l=1}^{K} y_{il}\left(\beta_{0l}+x_i^T\right)-\log\left(\sum_{l=1}^{K} e^{\beta_{0l}+x_i^T\beta_l}\right)-\lambda\sum_{l=1}^{K}\left(\alpha\|\beta_l\|_1+\frac{1}{2}(1-\alpha)\|\beta_l\|_2^2\right)\right]$$

Here, N is the number of samples and y_{il} is the class label that follows a one-of-K coding scheme (i.e equals 1 if subject i belongs to class ℓ and zero otherwise). The last term is an elastic-net penalty term where λ controls the overall amount of applied penalization and α specifies a balance between lasso and ridge penalty terms (i.e. the first and second terms in the penalty above). Logistic regression penalized with only lasso or ridge penalty is a special case of elastic-net, with $\alpha=1$ and $\alpha=0$ for pure lasso and pure ridge, respectively. The ridge part shrinks the coefficients whilst lasso part encourages variable selection (effectively forcing some coefficients to be exactly zero, and therefore removed from the model). Therefore, elastic net, similarly to lasso,[14] has can select only discriminating features. Elastic net, however, behaves better in the presence of highly correlated, informative groups of predictors. While lasso might select only one of the correlated variables, elastic net would select the whole group of correlated variables.

In our study we used a fixed value of $\alpha=0.2$ for all the analyses. While ultimately arbitrary, this value is closer to ridge regression than lasso, but it still contains its feature selection properties. That means it provides a reasonable balance in that it will select informative variables even if they are correlated with other variables in the model, thus minimizing the chance of overlooking variables that are informative, but that might be overshadowed by other, highly correlated, but slightly more informative variables.

Cross-validation

We used repeated 10 fold cross-validation to assess generalizability of the model. To achieve this, the dataset was divided in 10 subsamples, with approximately the same proportion of outcome classes in each subsample as in the full dataset. 9 subsamples served as a training set, and the remaining 10th served as a test set. The model was fit on the data from the training set and evaluated on the test set. This was repeated 10 times, so that every time a different subsample served as a test set, therefore each subsample served as a test set exactly once.

This whole procedure was repeated 10 times, with different splits of the data, in order to decrease dependence of the result on one particular data split. The final model performance was obtained by averaging results from the 100 test folds (10x 10-folds).

Optimal model parameters

In order to avoid double dipping and model selection bias, the optimal amount of regularization (λ) was selected for each fold separately, within additional (nested) 10-fold CV loop, performed on the training data only. Therefore, the selection of the amount of regularization applied was not influenced by the test data. CV was performed for 100 candidate λ values on a range between a value of λ that would result in a model containing only one predictor to a value of λ that would allow a model to keep all predictors as generated by the glmnet package.[13] λ that minimized cross-validated mean squared error (Brier score) was chosen to fit the model using the whole training set.

Performance measures

Discrete measures (e.g. sensitivity, balanced accuracy) were obtained by thresholding the probability predictions. In the binomial case (presence/absence of unipolar depression diagnosis at follow-up), the predictions were thresholded by class proportions, so that the class that was predicted with higher probability than the percentage of subjects in this class (i.e., class proportion) was selected. Using the same approach in the multinomial case (3 LCGA trajectory groups) can result in more than one predicted class for a participant and, therefore, we used a different thresholding procedure. Predicted probabilities for each class were normalized by the class proportions (probability of an individual belonging to a class X divided by the proportion of participants belonging to the class X) and the class with the highest normalized prediction was selected. Please note that when this method is applied in the binomial case it would produce the same result as the simple thresholding by class proportions approach.

Permutation testing

To establish whether the estimated models' performance were statistically significant, we repeated the whole procedure 1000 times with randomly permuted labels. The p-value was calculated as the proportion of permutations that achieved a higher AUROC than the ones obtained for the real labels.

Imputation of missing values

The mean value of 5 most similar individuals, measured by Euclidean distance, was imputed to replace each missing value. Only the data from the training set were used to compute this value. The proportion of missing data was small; in total 25 out of 80 variables contained missing values.

Of those variables, the median number of missing values per variable was 11 (1.4% of the sample). 23 out of 25 missing variables contained less than 7% of missing values with an exception of cortislol ROCg and cortisol ROCi with 38% of missing values.

Nonlinear and interaction analysis

We conducted additional exploratory analyses in order to model possible interaction and nonlinear age effects. We repeated the cross-validation and stability selection procedures for models with two additional sets of predictors: I) all variables and interactions between all pairs of variables. II) all variables, quadratic and cubic expansion of age, and interactions between all pairs of variables(including those with expanded age terms).

Stability selection

Additional separate analyses were performed in order to assess the statistical significance of individual predictors. For this purpose, we used a stability selection approach.[16] Stability selection combines feature selection methods such as elastic-net used here, with resampling, in order to find a set of features with a stable association with the outcome. The probability of a feature being selected is estimated by repeatedly fitting the model in different subsamples (without replacement) of the data. The estimated selection probability is then the proportion of subsamples in which the feature is selected. Since it is possible that in the case of multinomial logistic regression different features are selected for each of the binary models, the probability of a feature being selected was defined as the number of participants in which the feature was selected in at least one of the binary models. This selection probability can be computed along the range of possible penalization parameters and this is called a stability path. The stability path is a useful visualization tool, because it allows us to examine if a selected variable was consistently selected across wide range of regularization parameters.

The selected variables are variables that have higher probability of being selected than a pre-specified threshold. While the selection of this threshold is arbitrary, we used the default values suggested by the authors of the method,[15] which are justified because they lead to theoretical guarantees on bounding the number of falsely selected variables.[15] More specifically, stability selection allows us to compute an expected number of falsely selected variables at any given point in a stability path. This is used to specify a region in the stability path with a chance of falsely selected variables being smaller than a desired family-wise error rate (FWER).

Stability selection was performed using R package C06013. We used 1000 resamples of the default size of 63.2% of the full dataset, with the elastic-net logistic regression with $\alpha = 0.2$ as a feature selection method (same parameter value as used in our penalized logistic regression models) and a threshold of 0.75.

Supplemental results

IDS individual items

After identification of IDS as the only significant predictor of the course of depression, we performed the same stability selection analysis in order to identify which of the individual items from the IDS questionnaire were predictive of the course of depression. We identified only item "Feeling sad" to be significant for prediction of LCGA course trajectory classes and "Reactivity of mood" for diagnosis (presence/absence of MDD diagnosis at follow-up) prediction. AUROC's of models trained using only these two variables were 0.64 for predicting diagnosis and for LCGA groups: remission=0.68, improvement=0.61 and chronic=0.61.

Positive and negative predictive values

In addition to calculating the accuracy, sensitivity and specificity as an indication of the performance of our predictive models, we additionally calculated positive predictive values (PPV) and negative predictive values (NPV). The PPV is calculated as 'true positive/(true positives + false positives)' and the NPV is calculated as 'true negative/(true negative + false negative)'. Whereas sensitivity (true positive/(true positive + false negative)) indicates the probability for a person of being classified as belonging to e.g. the chronic course trajectory group if that person actually has a chronic course, the PPV indicates the probability of actually having a chronic course when a person is classified as such. Similarly, specificity (true negative/(true negative + false positive)) indicates the probability for a person of not being classified as belonging to e.g. the chronic course trajectory group if that person indeed didn't have a chronic course, whereas the NPV indicates the probability of not having a chronic course when a person is also not classified as such. The PPV and NPV are the proportions of positive and negative results of classification models that are true positive and true negative results, respectively.

The PPV and NPV provide important additional information, as they also take into account the prevalence of the group of interest (e.g. a chronic course trajectory). If, for example, the prevalence of the group of interest that is to be classified by the prediction model is low relative to the prevalence of another group that it is classified against, the PPV may be low even though the sensitivity can be high. Thus, a low PPV means that a high proportion of classified subjects are false positive classifications, which can have impact for the usefulness of the prediction model in clinical practice.

The PPV depends on an arbitrary selected classification threshold specifying what proportion of subjects from the whole sample will be classified as a specific course group. We present the PPVs in a graph across the whole range of possible proportions (Figure S1). In multinomial setting, PPV was assessed in a one-versus-all setting by collapsing multinomial probability predictions to three separate binomial predictions. For example, if the multinomial probabilities for class membership

for a specific subject were e.g. 0.2 for the Remission group, 0.3 for the Improvement group and 0.5 for the Chronic group, this would be converted into binomial probabilities of 0.2 for being classified as a member of the Remission group versus 0.8 of being classified into one of the other two, 0.3 versus 0.7 for Improvement group, and 0.5 versus 0.5 for the Chronic group. The same approach was applied for the NPVs and Figure S2 shows the NPVs across the whole range of possible proportions.

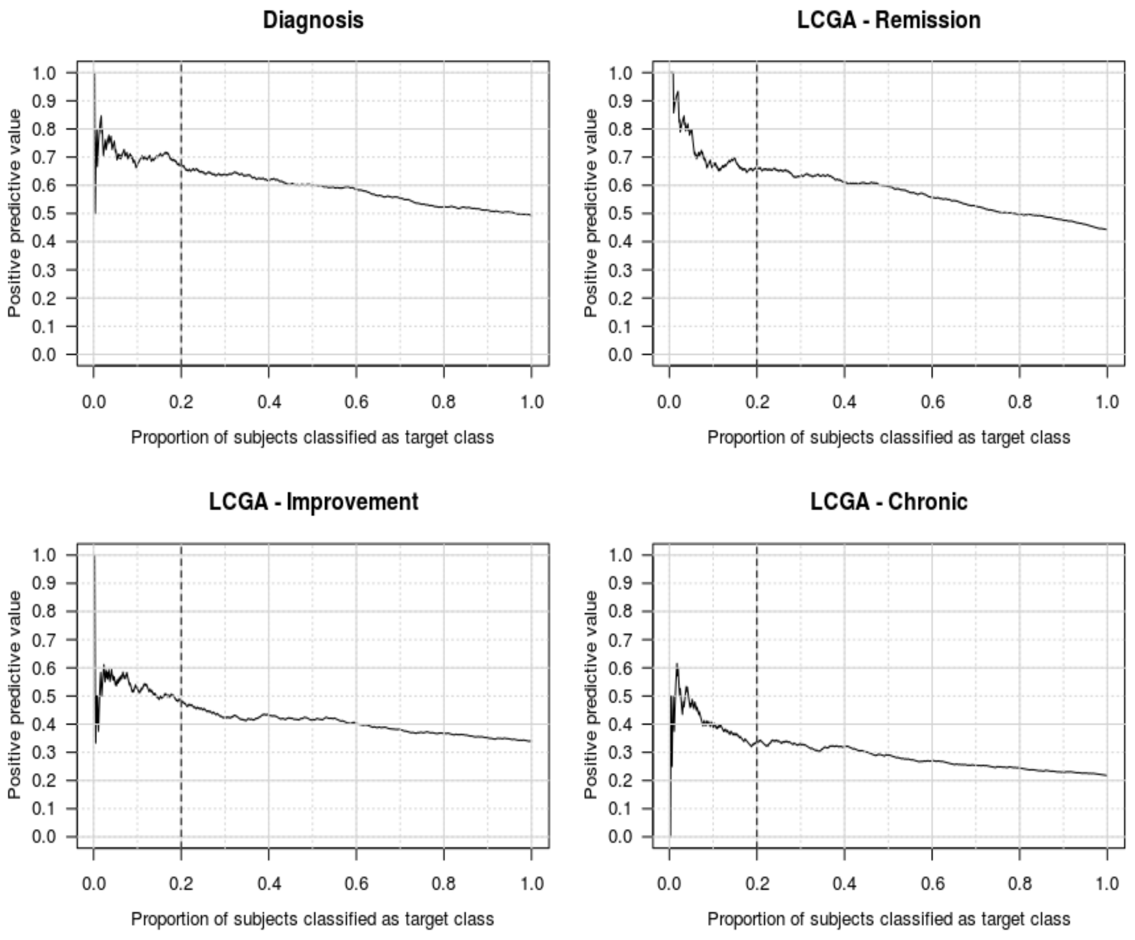

Figure S2: Positive predictive value (PPV) of the models with respect to percentage of the samples classified as a target class (course group). For example, for 20% of subjects with highest predicted chance to still have a depression diagnosis at 2-years follow-up, PPV was 0.68, and for 20% subjects with highest prediction of individual LCGA classes, PPV was 0.66 for remission, 0.5 for improvement and 0.33 for chronic course.

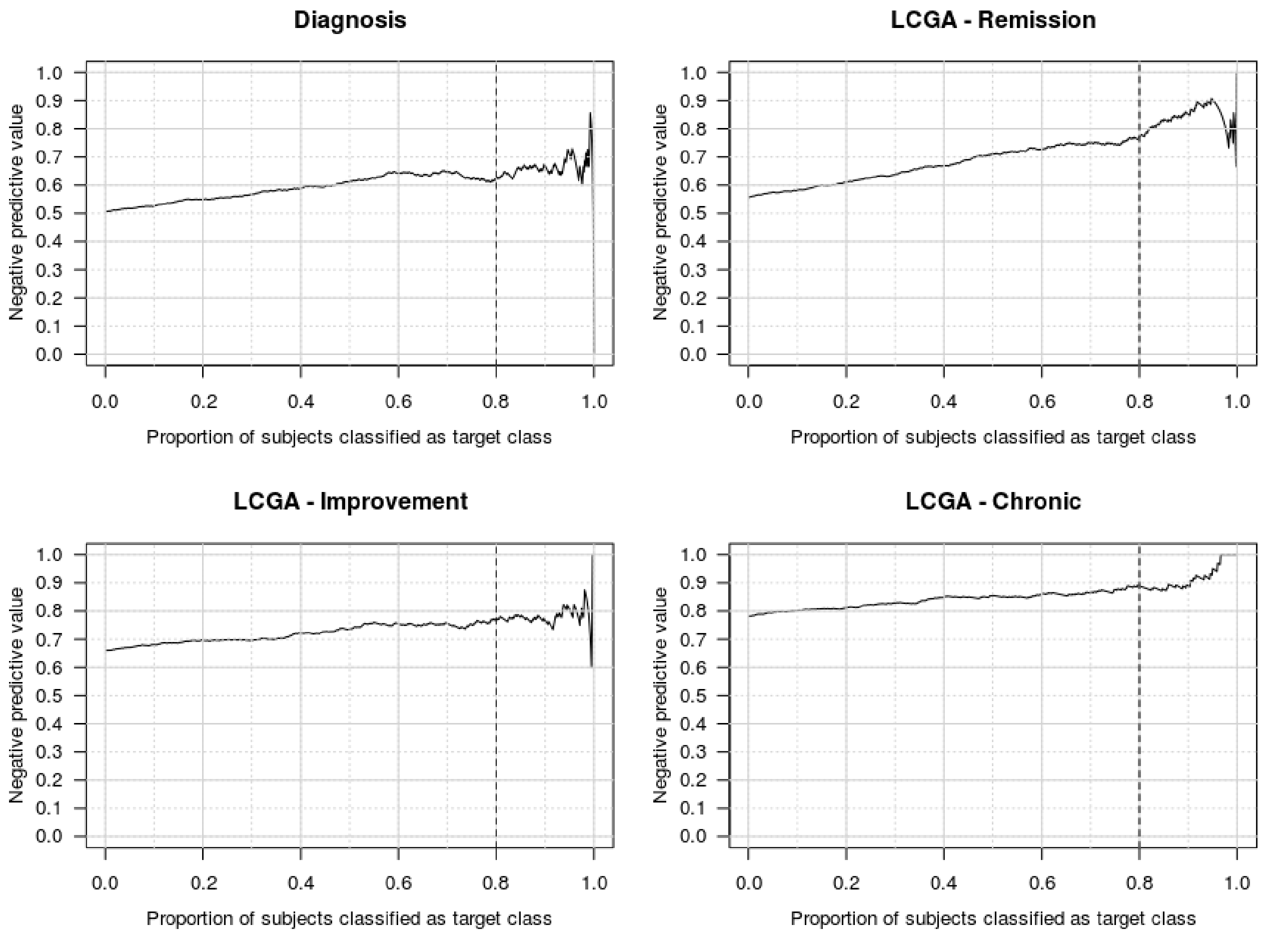

Figure S3: Negative predictive value (NPV) of the models with respect to percentage of the samples classified as a target class (course group).

Nonlinear and interaction analysis

AUROC's for models including all second order interaction terms (see main text) were 0.68 for REM, 0.58 for IMP, 0.61 for CHR, and 0.65 for prediction of MDD diagnosis at follow-up. AUROC's for models including quadratic and cubic expansions of age, and all second order interactions were 0.68 for REM, 0.57 for IMP, 0.62 for CHR, and 0.65 for prediction of MDD diagnosis.

Stability selection identified the total IDS score and conscientiousness (personality dimension) as statistically significant predictors of a unipolar depression diagnosis at follow-up in both additional expanded variable sets (a model including all second order interaction terms and a model including all second order interaction terms and quadratic and cubic expansions of age). Statistically significant predictors of LCGA course trajectories included the IDS total score, extraversion (personality dimension), recent dysthymia (in past month) and two-way interactions between one month and 6 months recency of dysthymia, and between one month and twelve months recency of dysthymia. The same variables were selected for both additional sets of predictors. The diagnoses of dysthymia with a one month, 6 month and 12 month recency variables are highly correlated and represent the same construct (dysthymia), therefore, the interaction between these diagnoses of dysthymia variables with different recencies are not very meaningful. All of the variables identified by stability selection for these additional exploratory models were amongst the highest ranked variables and selected by stability selection in the main analysis (models including only main effects), although variables other than the IDS total score did not survive FWER correction in the main analysis.

These results show that the AUROC's of the models including interaction terms and non-linear effects of age are similar to the AUROC's reported for models without these additional interaction terms and non-linear age terms. Thus, the nonlinear and interaction effects we included did not improve overall predictive performance for course prediction.

Used variables

Demographic, clinical, psychological and biological variables stratified by the outcome groups (presence of a diagnosis at follow-up or LCGA course trajectories). Binary or dummy-coded variables are shoed as bar charts. The rest is showed as boxplots.

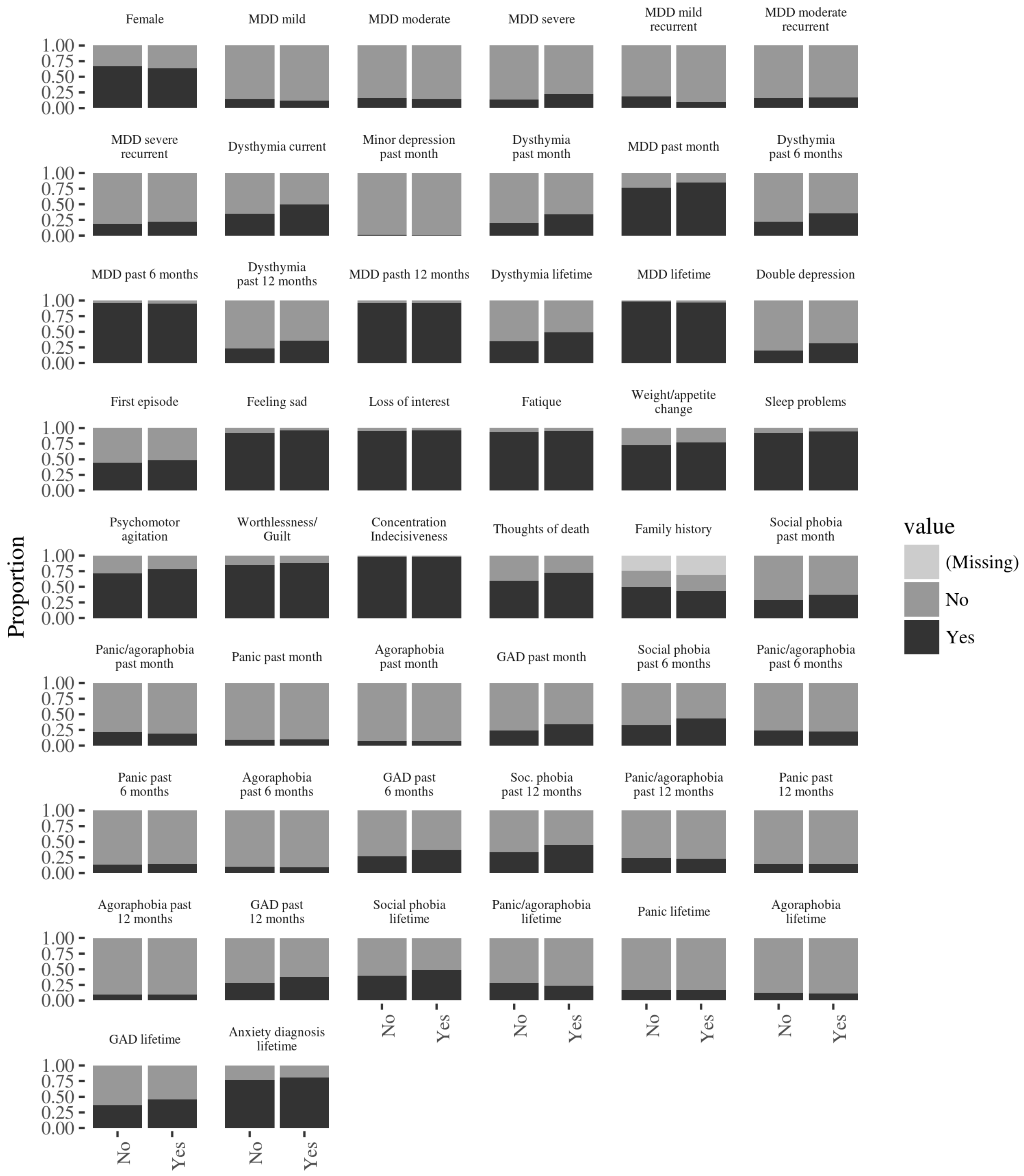

Figure S4: All used binary variables, stratified according to the outcome definition, the presence of a depression diagnosis two years after follow up. MDD: major depressive disorder, GAD: generalized anxiety disorder.

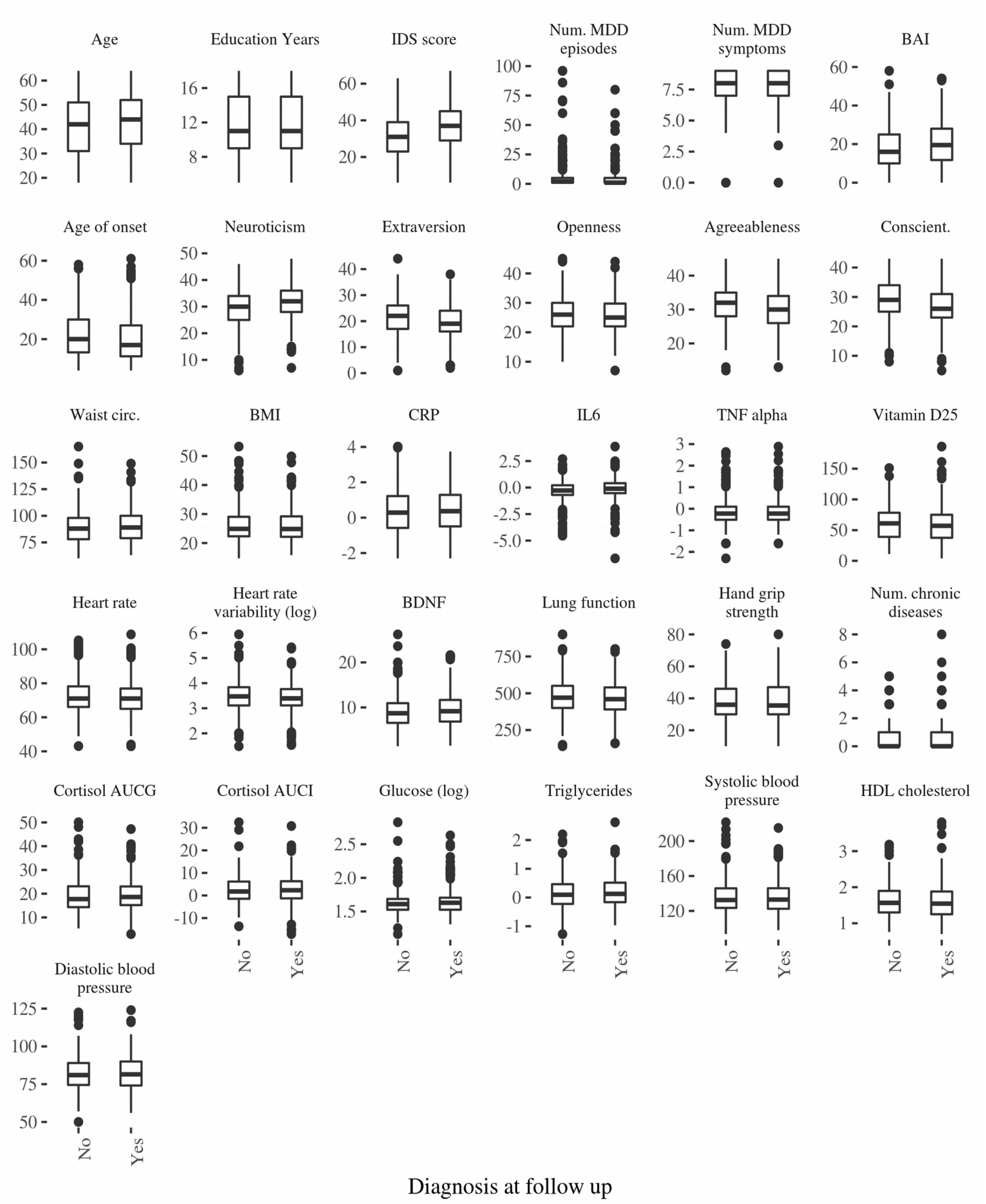

Figure S5: All used binary variables, stratified according to the outcome definition, the presence of a depression diagnosis two years after follow up. MDD: major depressive disorder, GAD: generalized anxiety disorder. Glucose and heart rate variability have been log transtformed. IDS: inventory of depressive symptomatology, BAI: Beck anxiety inventory, BMI: body-mass index, CRP: C-reactive protein, IL6: interleaukin 6, TNF alpha: tumor necrosis factor alpha, BDNF: brain derived neurotrophic factor.

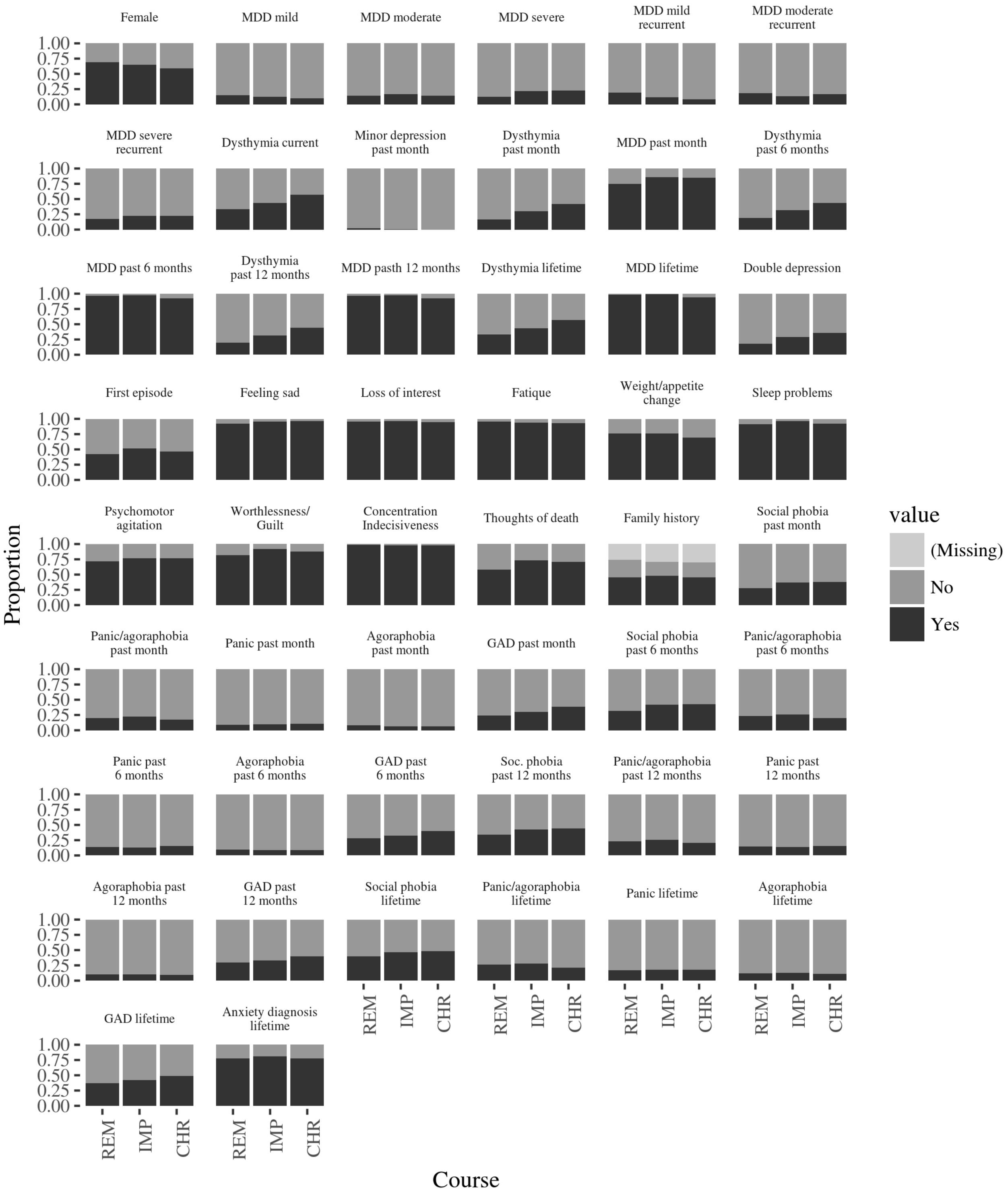

Figure S6: All used non-binary variables, stratified according to the 3 LCGA outcome classes: ramid remission (REM), gradual improvement (IMP) and chronic (CHR). MDD: major depressive disorder, GAD: generalized anxiety disorder.

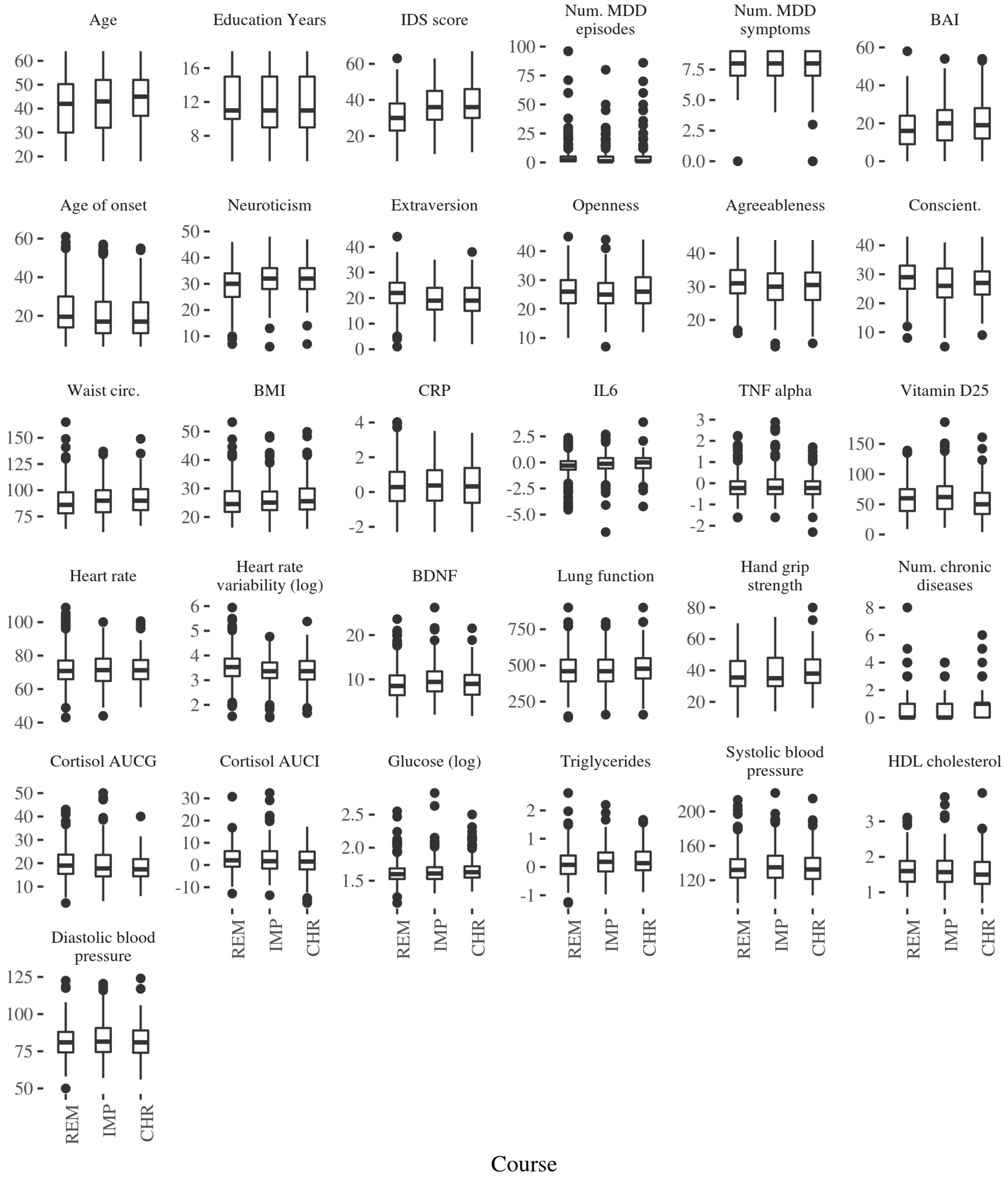

Figure S7: All used non-binary variables, stratified according to the 3 LCGA outcome classes: ramid remission (REM), gradual improvement (IMP) and chronic (CHR). MDD: major depressive disorder, GAD: generalized anxiety disorder. Glucose and heart rate variability have been log transtformed. IDS: inventory of depressive symptomatology, BAI: Beck anxiety inventory, BMI: body-mass index, CRP: C-reactive protein, IL6: interleaukin 6, TNF alpha: tumor necrosis factor alpha, BDNF: brain derived neurotrophic factor.

References

1. Rush AJ, Giles DE, Schlesser MA, Fulton CL, Weissenburger J, Burns C. The Inventory for Depressive Symptomatology (IDS): preliminary findings. *Psychiatry Res* 1986; 18: 65–87.
2. Beck AT, Epstein N, Brown G, Steer RA. An inventory for measuring clinical anxiety: Psychometric properties. *J Consult Clin Psychol* 1988; 56: 893–897.
3. de Graaf R, Bijl R V., Smit F, Vollebergh WAM, Spijker J. Risk Factors for 12-Month Comorbidity of Mood, Anxiety, and Substance Use Disorders: Findings From the Netherlands Mental Health Survey and Incidence Study. *Am J Psychiatry* 2002; 159: 620–629.
4. Wiersma JE, Hovens JGFM, Van Oppen P, Giltay EJ, Van Schaik DJF, Beekman ATF et al. The importance of childhood trauma and childhood life events for chronicity of depression in adults. *J Clin Psychiatry* 2009; 70: 983–989.
5. Fyer AJ, Weissman MM. Genetic linkage study of panic: clinical methodology and description of pedigrees. *Am J Med Genet* 1999; 88: 173–81.
6. Milaneschi Y, Hoogendijk W, Lips P, Heijboer AC, Schoevers R, van Hemert AM et al. The association between low vitamin D and depressive disorders. *Mol Psychiatry* 2014; 19: 444–451.
7. Vreeburg SA, Hoogendijk WJG, van Pelt J, DeRijk RH, Verhagen JCM, van Dyck R et al. Major Depressive Disorder and Hypothalamic-Pituitary-Adrenal Axis Activity. *Arch Gen Psychiatry* 2009; 66: 617.
8. van Milligen BA, Lamers F, de Hoop GT, Smit JH, Penninx BWJH. Objective physical functioning in patients with depressive and/or anxiety disorders. *J Affect Disord* 2011; 131: 193–199.
9. Schmaal L, Marquand AF, Rhebergen D, Van Tol MJ, Ruhé HG, Van Der Wee NJA et al. Predicting the Naturalistic Course of Major Depressive Disorder Using Clinical and Multimodal Neuroimaging Information: A Multivariate Pattern Recognition Study. *Biol Psychiatry* 2015; 78: 278–286.
10. Lyketsos CG, Nestadt G, Cwi J, Heithoff K, al et. The Life Chart Interview: A standardized method to describe the course of psychopathology. *Int J Methods Psychiatr Res* 1994.
11. Nylund, K. L., Asparouhov, T., Muthén, M. &, & Muthén, B. O. (n.d.). Deciding on the Number of Classes in Latent Class Analysis and Growth Mixture Modeling: A Monte Carlo Simulation Study. STRUCTURAL EQUATION MODELING, 14(4), 535–569.
12. Zou H, Hastie T. Regularization and variable selection via the elastic net. *J R Stat Soc Ser B Statistical Methodol* 2005; 67: 301–320.
13. Friedman J, Hastie T, Tibshirani R. Regularization Paths for Generalized Linear Models via Coordinate Descent. *J Stat Softw* 2010; 33: 1–22.

14. Tishirani R. Regression Shrinkage and Selection Via the Lasso. *J R Stat Soc Ser B* 1994; 58: 267-288.
15. Meinshausen N, Bühlmann P. Stability selection. *J R Stat Soc Ser B Statistical Methodol* 2010; 72: 417–473.
16. Sill M, Hielscher T, Becker N, Zucknick M. c060: Extended Inference with Lasso and Elastic-Net Regularized Cox and Generalized Linear Models. *J Stat Softw* 2014; 62: 1–22.
